# Data exploration of social client relationship management (CRM 2.0) adoption in the Nigerian construction business

**DOI:** 10.1016/j.dib.2018.04.037

**Published:** 2018-04-17

**Authors:** Rapheal A. Ojelabi, Adedeji O. Afolabi, Opeyemi O. Oyeyipo, Patience F. Tunji-Olayeni, Bukola A. Adewale

**Affiliations:** a Department of Building Technology, Covenant University, Nigeria; b Department of Quantity Surveying, Bells University of Technology, Nigeria; c Department of Architecture, Covenant University, Nigeria

**Keywords:** Client, Contractors, Construction industry, Relationship management, Social media

## Abstract

Integrating social client relationship management (CRM 2.0) in the built environment can enhance the relationship between construction organizations and client towards sustaining a long and lasting collaboration. The data exploration analyzed the e-readiness of contracting and consulting construction firms in the uptake of CRM 2.0 and the barriers encountered in the adoption of the modern business tool. The targeted organizations consist of seventy five (75) construction businesses operating in Lagos State which were selected from a pool of registered contracting and consulting construction firms using random sampling technique. Descriptive statistics of the e-readiness of contracting and consulting construction firms for CRM 2.0 adoption and barriers limiting its uptake were analyzed. Also, inferential analysis using Mann–Whitney U statistical and independent sample t-test was performed on the dataset obtained. The data generated will support construction firms on the necessity to engage in client social relationship management in ensuring sustainable client relationship management in the built environment.

**Specifications table**

**Table:** 

Subject area	Construction Management.
More specific subject area	Relationship Management.
Type of data	Tables and Figures
How data was acquired	Cross-sectional Survey design
Data format	Raw, analyzed.
Experimental factors	Random sampling of Construction organizations
Experimental features	Readiness and Barriers to Client Social Relationship Management (CRM 2.0) adoption by construction organizations
Data source location	Lagos, Nigeria.
Data accessibility	*All the data are in this data article*

**Value of the data**

• Clients are the most important entity in the construction business, therefore managing their needs is paramount to the success of construction organizations.
• The dataset will enable researchers to advance on the subject of social client relationship management in the built environment as there is a dearth of studies in this area as it relate to the construction industry [1], [2], [3], [4].
• An understanding of the data when analyzed compared with existing data on client relationship management can help ascertain the sustainable route to managing client relationship in the built environment.
• The dataset can help construction stakeholders understand the barriers militating against the uptake of client social relationship management so as to develop a framework that can successfully increase the uptake of ICT tools and other relationship management tools in the built environment.
• Clients are the most important entity in the construction business, therefore managing their needs is paramount to the success of construction organizations.

## 1 Data

Social client relationship management (CRM 2.0) is a business strategy that uses internet and social media platforms to enhance traditional, one-dimensional interactions between companies and their existing and potential customers by giving clients greater control over how they communicate with the firms they do business with and providing them with the tools needed to form the foundation of the relationship. In order to measure the relationship management that exist between clients and construction organization, the data exploration covered contractor and consultant construction firm's readiness to uptake social client relationship management (CRM 2.0) in the built environment and barriers militating against its uptake. The necessity of the data on Client Relationship Management uptake is due to the slow rate of adoption of the modern client management tool in the built environment compared to other industries like telecommunication and logistic enterprise [1], [2], [3], [4]. A questionnaire instrument was retrieved from seventy five (75) construction organizations which included contracting and consulting firms. The data focused on the presence and use of different social platforms by construction firms as shown in Table 1. In Table 1, the overall mean score showed that social media presence is mostly maintained by contracting and consulting firms via personal websites owned by the firm, Facebook and LinkedIn. This means that efforts by the construction business to reach out to clients in order to manage their relationships are attained using these social media platforms. The data generated is necessary due to the relevance of the social platforms in the modern client relationship management. Likewise, data are garnered on the barriers militating against the uptake of the social client relationship management (CRM 2.0) which is presented in Table 2. In Table 2, the overall mean score revealed that the most significant barriers encountered by construction businesses in the use of social client relationship management (CRM 2.0) include lack of business strategy by organizations, Lack of control over social media use and size of the construction organizations. The dataset revealed that construction business must consciously and judiciously draft a business strategy that includes relationship management in order to survive the construction terrain. When the data is analyzed, further inferential statistical decisions can be made from the data exploration. Inferential statistics such as Mann–Whitney U test which measured if there was any significant difference between contracting and consulting firms on the use of social media platforms for social client relationship management was tested (Table 3, Table 4, Table 5). In Table 4, the p-value at .601 showed that there was no significant difference between contracting and consulting firms on the use of social media platforms for social client relationship management. This means that both contracting and consulting firms aligned in the social media platforms used for social client relationship management in their construction business. Furthermore, independent sample t-test was conducted to ascertain if there was significant difference between contracting and consulting firms on barriers militating against the adoption of social media in social client relationship management (CRM 2.0) as presented in Table 6, Table 7. In Table 7, the *p*-value at .40 and .36 which is higher that the alpha value of .05 depicts that there was no significant difference between contracting and consulting firms on barriers militating against the adoption of social media in social client relationship management (CRM 2.0) in their construction business.

**Table 1:** Social media presence of construction organizations.

**Social media platforms**	**Contracting firms**	**Consulting firms**	**Overall mean score**
	**Mean score**	**Mean score**	
Company's own website	3.41	3.31	3.43
Facebook	3.00	3.62	3.28
LinkedIn	2.87	3.34	3.00
Google+	2.70	2.90	2.73
WordPress	2.50	2.48	2.48
Instagram	2.22	2.07	2.16
Twitter	1.93	1.62	1.86
Social bookmarking sites	1.67	1.55	1.63
YouTube	1.57	1.62	1.59
Blogger	1.35	1.24	1.32
Snapchat	1.28	1.38	1.32
Pinterest	1.26	1.62	1.38
Flickr	1.24	1.24	1.24
Yammar	1.17	1.21	1.18
Vimeo	1.13	1.28	1.18

**Table 2:** Barriers to organizations readiness for CRM 2.0 adoption.

**Barriers**	**Contracting firm**	**Consulting firm**	**Overall mean score**
	**Mean Score**	**Mean Score**	
Lack of business strategy by organizations	2.65	3.10	2.82
Lack of control over social media use	2.70	2.96	2.81
Construction organizations size	2.54	3.03	2.77
Managements unwillingness to adopt new technology	2.54	3.03	2.77
Complex nature of client-organization relationship	2.59	2.96	2.76
Organizations lack of investment on social software management tools	2.70	2.75	2.72
Organizations lack of knowledge of social media client management capacity in the built environment.	2.60	2.79	2.71
Managements negative perception about social platforms.	2.35	2.82	2.54
External pressure from competitors	2.45	2.42	2.49
Fear of clients information leakages by social platforms managers	2.36	2.46	2.41

**Table 3:** Mean ranks of differences in contracting and consulting organizations use of social media for CRM 2.0.

	Organization type	*N*	Mean rank	Sum of ranks
Social Media Platform	contracting	46	36.96	1700.00
	consulting	29	39.66	1150.00
	Total	75

**Table 4:** Mann–Whitney U test statistics of differences in contracting and consulting organizations use of Social media for CRM 2.0.

	Social media platform
Mann–Whitney U	619.000
Wilcoxon W	1700.000
Z	−.523
Asymp. Sig. (2-tailed)	.601

**Table 5:** Median score of differences in contracting and consulting organizations use of social media for CRM 2.0.

Organization type	*N*	Median
Contracting	46	26.0000
Consulting	29	30.0000
Total	75	27.0000

**Table 6:** Mean rank of difference in contracting and consulting construction organizations on the barriers to CRM 2.0 adoption.

	Organization type	*N*	Mean	Std. deviation	Std. error mean
Challenges	Contracting	46	25.5000	5.90198	.87020
	Consulting	28	28.3571	5.35561	1.01211

**Table 7:** Independent sample *t*-test on difference in contracting and consulting construction organizations' perception on barriers to social media adoption in CRM 2.0.

		Barriers			
		Equal variances assumed		Equal variances not assumed	
		(Upper)	(Lower)	(Upper)	(Lower)
Levene's Test for equality of variances	F		.225		
	Sig	.637			
*t*-Test for equality of means	T		−2.090		−2.141
	Df	72		61.506	
	Sig. (2 tailed)		.40		.36
	Mean difference		−2.857		−2.857
	Standard error difference	1.367		1.334	
	95% Confidence interval of the difference	−.132	−5.582	−.188	−5.526

## 2 Experimental design, materials and methods

The population for the data exploration is the summation of construction organizations which comprises of consulting and contracting firms in Nigeria. The dataset collected is more specific to construction business of contracting and consulting firms operating in Lagos State. Lagos State has many head offices of construction organizations within the state. The contracting and consulting construction firms used to generate the data were selected randomly from the pool of the record of registered construction organizations in the study area. A cross-sectional research survey design was used in selecting the sample size of seventy-five (75) construction organizations. Questionnaire instruments were directed to managerial staff that deal with client and client organizations. Similar field surveys that have obtained dataset in like manner include [5], [6], [7], [8], [9], [10], [11], [12], [13], [14], [15], [16], [17], [18]. In future studies, the client or client organizations' perspective can be measured as regards the effectiveness of contracting and consulting firms to the issues raised on construction projects. The dataset can be replicated in other climes and compared with analysis in this data exploration.
